# Clinical and radiologic outcomes in patients undergoing primary total hip arthroplasty with Collum Femoris Preserving stems: a comparison between the direct anterior approach and the posterior approach

**DOI:** 10.1186/s12891-022-05040-2

**Published:** 2022-01-22

**Authors:** Bingshi Zhang, Sikai Liu, Zeming Liu, Bo Liu, Jia Huo, Mengnan Li, Yongtai Han

**Affiliations:** Department of Osteonecrosis and Hip Surgery, The Third Hospital of Hebei Medical University, No. 139 Ziqiang Road, Shijiazhuang, Hebei People’s Republic of China

**Keywords:** Direct anterior approach, Posterior approach, Collum femoris preserving, Total hip arthroplasty, Outcomes

## Abstract

**Background:**

The direct anterior approach (DAA) and posterior approach (PA) are two of the most common surgical approaches used for total hip arthroplasty (THA) worldwide. The curved anatomical collum femoris-preserving (CFP) stem was designed to preserve the bone of the femoral neck and allow physiologic load transfer along the trabecular systems, which may better restore hip biomechanics, improve triplanar stem stability and improve the long-term survival rate. We believe this study will demonstrate whether the DAA is suitable for THA with CFP stems.

**Methods:**

The data of patients who underwent primary THA with CFP stems from January 2010 to December 2015 were retrospectively analysed. These patients were divided into two groups (group A, DAA; group B, PA). The approach was selected by the surgeon. The demographic characteristics, preoperative diagnoses, preoperative Harris hip score, preoperative range of motion, postoperative complications, and radiologic measurements (neck-shaft angle, coronal alignment, sagittal alignment, stress shielding, anteversion, neck-preserving ratio, acetabular anteversion, acetabular inclination angle, acetabular depth, anterior-posterior offset, lateral offset, difference in leg length) were recorded for all patients.

**Results:**

In this study, a total of 248 patients (185 male and 63 female) were included. No significant differences were found between group A and group B in terms of general patient information and radiologic measurements. However, the rate of nerve injury in group A (7/5.5%) was significantly higher than that in group B (1/0.8%) (*p* = 0.037). At 1 month after surgery, we found a significant difference between the two groups in the Harris hip score (HHS) (71.03 ± 8.04 in group A, 68.39 ± 8.37 in group B, *P* = 0.014) and forgotten joint score (FJS-12) (50.78 ± 7.57 in group A, 47.68 ± 7.34 in group B, *P* = 0.001). At 1 year after surgery, the mean FJS-12 score in group A (68.78 ± 7.54) was higher than that in group B (58.84 ± 8.91) (*P* < 0.001). At 5 years after surgery, the mean FJS-12 score in group A (73.38 ± 7.21) was higher than that in group B (67.16 ± 9.12) (P < 0.001). Post hoc analysis of the 1-month, 1-year, and 5-year postoperative FJS-12 scores using multiple linear regression analysis revealed that an excellent HHS led to good patient satisfaction at each time point.

**Conclusion:**

In summary, unlike a “standard” femoral stem, whose alignment might be affected by the surgical approach, alignment of the CFP stem is independent from the surgical approach. Even though the DAA had a higher nerve injury rate, nerve injury from the DAA did not typically cause severe dysfunction of the lower extremity. Therefore, decisions regarding the surgical approach for patients undergoing THA with CFP stems can be made primarily based on the preference of the surgeon.

## Introduction

Total hip arthroplasty (THA) is considered one of the most successful surgical procedures because it relieves pain, restores mobility, and improves quality of life in patients who previously had incapacitating arthritis and osteonecrosis. The surgical approach may influence postoperative recovery and the rates of complications after THA. In recent years, the orthopaedic community has explored different surgical approaches for THA [1–6]. The direct anterior approach (DAA) and posterior approach (PA) are 2 of the most common surgical approaches used for THA worldwide. Surgeons have attempted to reduce the rate of complications and increase the speed of postoperative recovery after primary THA with the use of the DAA. Compared with the PA, the DAA has been shown to yield lower pain scores, earlier functional recovery, and a lower postoperative dislocation rate [7–11]. A meta-analysis conducted by Miller et al. [12] revealed a shorter incision length, less severe pain experienced in the hospital, a lesser need for opioid medications and a shorter length of stay in the DAA group than in the PA group. However, these previous studies did not use all the types of cementless femoral stems.

**Fig. 1:**
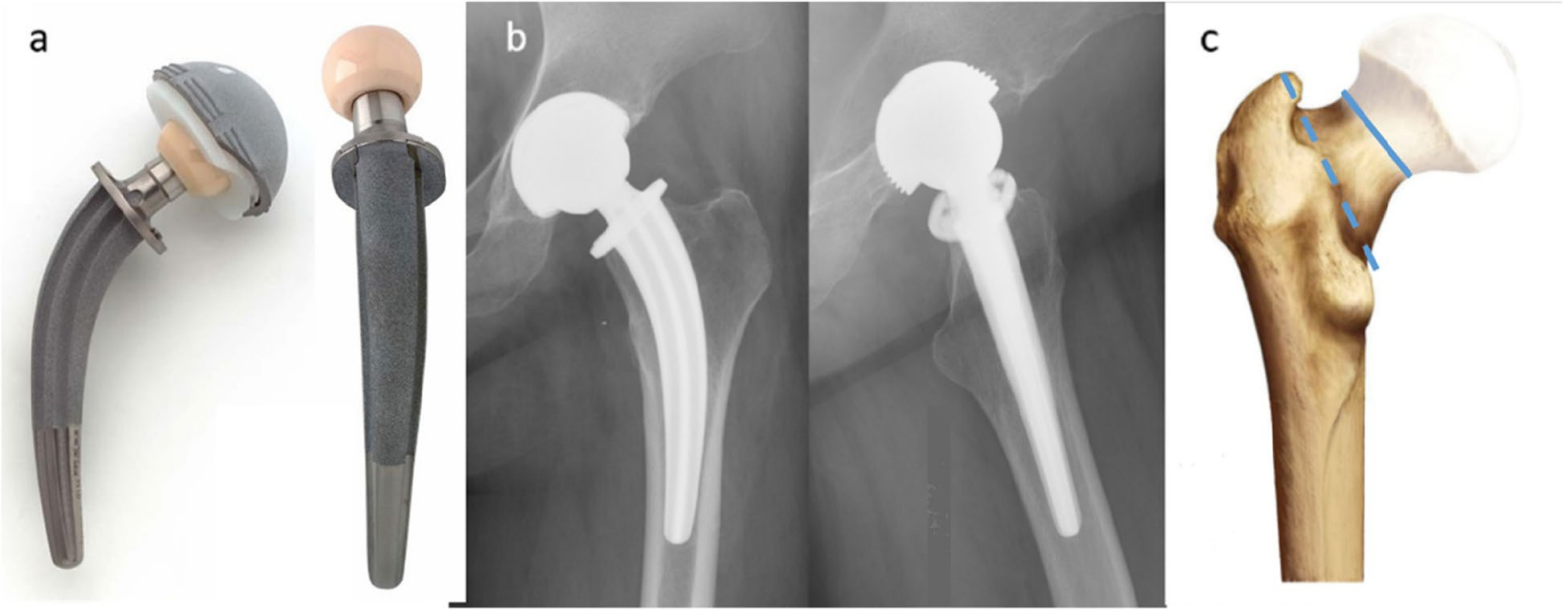
Characteristics of CFP prostheses. **a**. The geometry of the collum femoris-preserving stem (Waldemar-Link, Hamburg, Germany) can be characterized as a curvature design and self-anteversion. **b**. Postoperative anteroposterior and lateral radiographs of a 57-year-old female with left osteonecrosis of the femoral head. c. The osteotomy position of CFP stem is indicated by the solid line, and the traditional neck resection stem is indicated by the dotted line. The femoral neck between these two lines is preserved during hip arthroplasty when the CPF stem is implanted

It has been confirmed that stem design is related to the presence of early aseptic loosening in cementless THA. Femoral stems with an angular shoulder are associated with an increased risk of aseptic loosening, and anatomically shaped stems are suggested to be used when DAA THA is performed [13]. The curved anatomical collum femoris-preserving stem (Fig. 1a, b) was designed to preserve the bone of the femoral neck and allow physiologic load transfer along the trabecular systems by distributing the stresses towards the medial lateral diaphysis, thereby better restoring hip biomechanics, improving triplanar stem stability, and improving the long-term survival rate [14]. Furthermore, when the femoral neck is preserved, the osteotomy level is closer to the femoral head than it is without femoral neck preservation. In DAA THA, this preserved femoral neck can make it easier to implant the stem in the medullary cavity of the femur. A large number of studies have confirmed that PA THA with the CFP stem is a good option for patients who need to undergo prosthesis revision and has satisfactory long-term effectiveness [15]. However, almost no studies have explored the mid- or long-term clinical and radiologic outcomes of DAA THA with CFP stems. Moreover, the clinical and radiologic outcomes of the DAA and PA for THA with a single CFP stem have not been compared. In this study, we collected the data of patients who underwent THA with CFP stems with either approach. The patients were divided into two groups according to the approach used, and the data were retrospectively reviewed. The study was conducted to reveal whether the DAA is suitable for CFP stems.

## Materials and methods

### Study population

The data of patients who underwent primary THA with CFP (Waldemar-Link, Hamburg, Germany) stems from January 2010 to December 2015 were retrospectively analysed in this study. Patients with stage III or IV unilateral osteonecrosis of the femoral head (ONFH) according to the Association Research Circulation Osseous classification system, grade I to III developmental dysplasia of the hip (DDH) according to the Crowe classification system, grade III or IV osteoarthritis according to the Kellgren–Lawrence classification system or other end-stage hip diseases (such as necrosis of the femoral head after femoral neck fracture or periacetabular fractures) were enrolled. Patients with malignant diseases, metabolic bone diseases, bacterial inflammation, or incomplete medical records or radiologic images were excluded [14]. The patients were divided into two groups according to the approach used (group A, DAA; group B, PA). The approach was selected by the surgeon. The study was approved by the Institutional Review Board of the Third Hospital of Hebei Medical University and was conducted in accordance with the Declaration of Helsinki and regulations of the Health Insurance Portability and Accountability Act (HIPAA). Because this was a retrospective study and the data were anonymous, the requirement for informed consent was waived after being granted by the Ethics Committee of the Third Hospital of Hebei Medical University.

### Surgical technique and postoperative treatment

All operations were performed by the same group of surgeons who had experience performing both approaches. Preoperative templating was not routinely performed for any patient. A subcapital osteotomy was made in all patients.

**Fig. 2:**
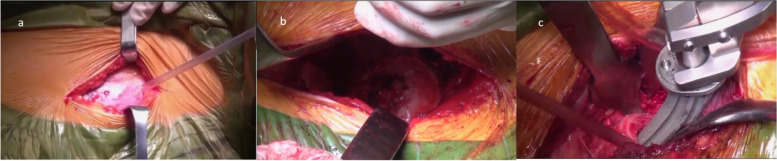
The implantation process of a CFP stem via the direct anterior approach (DAA). **a**. Incision for the direct anterior approach. **b**. Exposure of the acetabulum in the direct anterior approach. **c**. Femoral exposure and stem implantation in the direct anterior approach

The procedure used in the DAA group during the study period followed the surgical technique described by Nakata et al. and Lovell et al. [16, 17]. A standard operative table was used. After the tensor fasciae lata and sartorius were retracted, the anterior aspect of the hip capsule was exposed by displacing the gluteus minimus and the rectus femoris muscle. The anterior aspect of the hip capsule was routinely removed. To expose the proximal femur and elevate it slightly above skin level to allow access to the femoral canal, the superior capsule was released (Fig. 2).

**Fig. 3:**
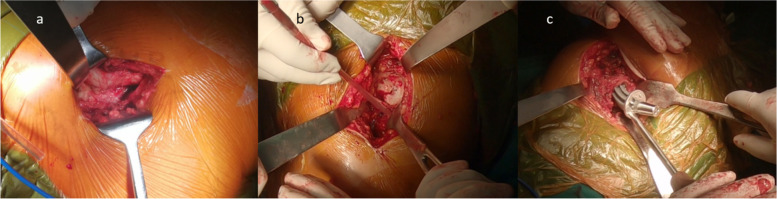
The implantation process of a CFP stem via the posterior approach (PA). **a**. Incision for the posterior approach. **b**. Exposure of the acetabulum in the posterior approach. **c**. Femoral exposure and stem implantation in the posterior approach

In the PA group, the posterior joint capsule was removed, and the incised short external rotators were reattached to the greater trochanter (Fig. 3).

### Outcomes

Demographic characteristics and clinical parameters such as sex, age, body mass index (BMI), alcohol consumption status, smoking status, affected side, preoperative diagnosis, preoperative total HHS, range of motion and postoperative complications were recorded for all patients.

In all patients, anteroposterior view and lateral view X-ray examinations for the proximal femur were performed before and after the operation. All radiologic measurements (neck-shaft angle, coronal alignment, sagittal alignment [18], stress shielding [19], anteversion, neck-preserving ratio, acetabular anteversion, acetabular inclination angle, acetabular depth, anterior-posterior offset, lateral offset, difference in leg length) were independently performed by 2 experienced orthopaedic surgeons using data obtained from the picture archiving and communication systems of our hospital, and the values from the two surgeons were averaged. To asses intra - and interobserver repeatability, 20 patients were randomly selected, and each measurement was independently measured twice at an interval of 1 week. All intraclass correlation coefficients used to evaluate reproducibility in this study were > 0.9. The clinical variables assessed during this study included the FJS-12 scores and Harris scores at 1 month, 1 year, and 5 years postoperatively.

### Statistical analysis

SPSS version 19 (IBM SPSS Statistics for Windows) was used to analyse the data. All data were entered into a database created for statistical analysis. Continuous variables are expressed as the mean ± standard deviation. The chi-square test was used to compare the categorical variables. The Shapiro-Wilk test was used for data normality, and Levene test was used for homogeneity of variance. Non-parametric tests were used for comparisons between groups. Multiple linear regressions were used to account for the significant differences in the demographic data found between the 2 cohorts. *P*-values < 0.05 were considered significant [14].

## Results

### General information

In this study, a total of 248 patients (185 male and 63 female) were included. The mean age at surgery was 52.00 ± 12.42 years in group A and 51.15 ± 11.88 years in group B, and the difference between the groups was not significant (*P* = 0.575). There was also no difference between group A and group B in BMI (25.45 ± 3.47 kg/m^2^ vs 24.82 ± 3.71, *P* = 0.088). The smoking and alcohol statuses are shown in Table 1. In terms of indications for THA, 225 patients had ONFH, which was alcohol induced in 98 patients (53 in the DAA group, 45 in the PA group), glucocorticoid related in 53 patients (25 in the DAA group, 28 in the PA group), idiopathic in 71 patients (39 in the DAA group, 32 in the PA group), and due to other causes in 3 patients (2 in the DAA group, 1 in the PA group); 10 patients had DDH; 4 patients had osteoarthritis; and 9 patients had other end-stage hip diseases, such as ankylosing spondylitis with hip stiffness, necrosis of the femoral head after femoral neck fracture or acetabular fracture.

**Table 1:** General information of the patients in the two groups

General characteristics	Group A (*N* = 127)	Group B (*N* = 121)	Test statistics	*P*
Age (years)	52.00 ± 12.42	51.15 ± 11.88	0.561^#^	0.575
Sex				
Male	94	91	0.046^*^	0.830
Female	33	30		
BMI (kg/m^2^)	25.45 ± 3.47	24.82 ± 3.71	1.719^#^	0.088
Smoking status				
Yes	16	15	0.002^*^	0.962
No	111	106		
Alcohol consumption				
Yes	20	15	0.574^*^	0.449
No	107	106		
Indications				
ONFH	119	106	1.988^*^	0.575
DDH	4	6		
OA	1	3		
Other	3	6		
Preoperative HHS	51.39 ± 11.94	52.47 ± 10.47	1.138^#^	0.255
Range of motion (°)				
Flexion-extension	90.13 ± 17.34	88.84 ± 17.80	0.540^#^	0.589
Abduction-adduction	50.31 ± 13.53	49.22 ± 14.22	0.685^#^	0.494
External rotation-internal rotation	45.48 ± 16.11	47.74 ± 15.93	0.905^#^	0.365

Note: Group A: DAA, group B: PA,

BMI, body mass index; ONFH, osteonecrosis of the femoral head; DDH, developmental dysplasia of the hip; OA, osteoarthritis; “other” includes fractures of the femoral neck and femoral head necrosis after acetabular surgery

*Chi-square test, ^#^Mann-Whitney U test

The function and range of motion (ROM) of the affected hip was also investigated for each patient. The preoperative average HHS was 51.39 ± 11.94 in group A and 52.47 ± 10.47 in group B (*p* = 0.255). The preoperative flexion-extension ROM was 90.13 ± 17.34 in group A and 88.84 ± 17.80 in group B (*P* = 0.589). The preoperative abduction-adduction ROM was 50.31 ± 13.53 in group A and 49.22 ± 14.22 in group B (*P* = 0.494). The preoperative external rotation-internal rotation ROM was 45.48 ± 16.11 in group A and 47.74 ± 15.93 in group B (*P* = 0.365). The indications for the two groups are shown in Table 1.

### Radiological and clinical outcomes

The radiologic indicators were divided into two groups: femoral component position indicators and acetabular component position indicators. In terms of femoral component position indicators, no significant differences were found between group A and group B in the neck-shaft angle (132.86 ± 4.79° vs 133.35 ± 5.04°, Z = 0.923, *P* = 0.356), anteversion (13.12 ± 2.55° vs 13.16 ± 2.62°, Z = 0.092, *P* = 0.927), neck-preserving ratio (65.44 ± 8.69% vs 66.05 ± 7.37%, Z = 0.408, *P* = 0.683), anterior-posterior offset (48.81 ± 6.79 mm vs 47.53 ± 5.69 mm, Z = 1.644, *P* = 0.100), lateral offset (15.02 ± 6.25 mm vs 15.34 ± 6.57 mm, Z = 0.364, *P* = 0.716) and difference in leg length (3.50 ± 2.79 mm vs 2.85 ± 2.45, Z = 1.880, *P* = 0.060) . In group A, there were 121 femoral stems in the coronal neutral position, 4 stems in the varus position, and 2 stems in the valgus position. In group B, there were 112 femoral stems in the coronal neutral position, 6 stems in the varus position, and 3 stems in the valgus position. No significant differences were observed between the two groups (*P* = 0.668). In group A, there were 116 femoral stems in the sagittal neutral position, 8 stems in the flexed position, and 3 stems in the extended position. In group B, there were 114 femoral stems in the sagittal neutral position, 5 stems in the flexed position, and 2 stems in the extended position. No significant differences were observed between the two groups (*P* = 0.680). The BMD change ratio in Gruen zone 1 at the last follow-up was 0.90 ± 0.69 for group A and 0.89 ± 0.79 for group B (*P* = 0.167). The BMD change ratio in Gruen zone 7 at the last follow-up was 0.85 ± 0.79 for group A and 0.87 ± 0.75 for group B (*P* = 0.079).

In terms of acetabular component position, no significant differences were found between group A and group B in acetabular anteversion (26.61 ± 13.9° vs 28.41 ± 14.59°, Z = 1.132, *P* = 0.257), acetabular inclination angle (48.23 ± 5.83° vs 47.52 ± 5.69°, Z = 0.520, *P* = 0.630), and acetabular depth (2.97 ± 0.56 mm vs 2.91 ± 0.65 mm, Z = 0.586, *P* = 0.558) (Table 2).

**Table 2:** Radiological indicators of the prosthesis position for the two groups (postoperatively)

Radiological indicators	Group A (N = 127)	Group B (N = 121)	Z	*P*
Neck-shaft angle (°)	132.86 ± 4.79	133.35 ± 5.04	0.923^b^	0.356
Coronal alignment			0.807^a^	0.668
Neutral	121 (95.3%)	112 (92.6%)		
Varus	4 (3.1%)	6 (5.0%)		
Valgus	2 (1.6%)	3 (2.5%)		
Sagittal alignment			0.772^a^	0.680
Neutral	116 (91.3%)	114 (94.2%)		
Flexed	8 (6.3%)	5 (4.1%)		
Extended	3 (2.4%)	2 (1.7%)		
Stress shielding (BMD change ratio)				
Gruen zone 1	0.90 ± 0.69	0.89 ± 0.79	1.382^b^	0.167
Gruen zone 7	0.85 ± 0.79	0.87 ± 0.75	1.758^b^	0.079
Anteversion (°)	13.12 ± 2.55	13.16 ± 2.62	0.092^b^	0.927
neck-preserving ratio (%)	65.44 ± 8.69	66.05 ± 7.37	0.408^b^	0.683
Acetabular anteversion (°)	26.61 ± 13.99	28.41 ± 14.59	1.132^b^	0.257
Acetabular inclination angle (°)	48.23 ± 5.83	47.52 ± 5.69	0.520^b^	0.630
Acetabular depth (mm)	2.97 ± 0.56	2.91 ± 0.65	0.586^b^	0.558
Anterior-posterior offset (mm)	48.81 ± 6.79	47.53 ± 5.69	1.644^b^	0.100
Lateral offset (mm)	15.02 ± 6.25	15.34 ± 6.57	0.364^b^	0.716
Difference in leg length (mm)	3.50 ± 2.79	2.85 ± 2.45	1.880^b^	0.060

Note: Group A: DAA, group B: PA

^a^Chi-square test, ^b^Mann-Whitney U test

**Fig. 4:**
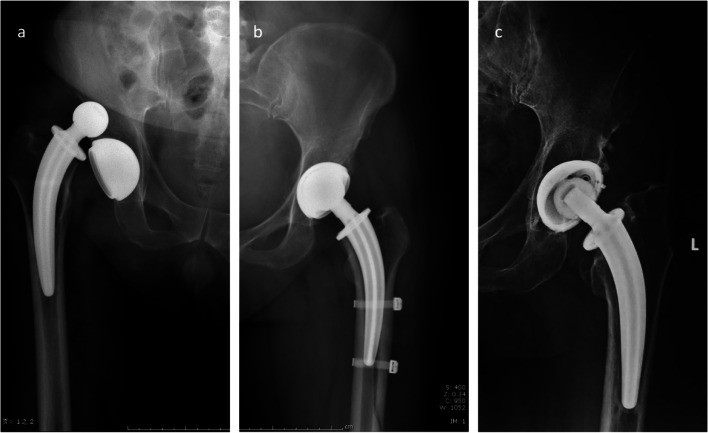
Typical complications of CFP stems in the two groups. **a**. In the PA group, a 50-year-old male suffered from prosthesis dislocation 3 months after the operation. **b**. In the DAA group, intraoperative periprosthetic femoral fracture occurred in a 58-year-old female with left acetabular dysplasia. The operator treated the fracture by using a cerclage cable. **c**. A 65-year-old male patient in the PA group with left osteonecrosis of the femoral head. This patient developed left hip pain 4 years after THA. Aseptic loosening of the prosthesis was diagnosed after radiographic examination

There were 8 patients (3.2%) with periprosthetic femoral fractures (PFFs), 7 patients (2.8%) with dislocations, 8 patients (3.2%) with nerve injuries (lateral femoral cutaneous nerve in DAA and sciatic nerve in PA), 14 patients (5.7%) with thigh pain and 2 patients (0.8%) with aseptic loosening (Fig. 4). The total prevalence of complications was 14.9%. The prevalence of complications in each group was also analysed (Table 3). There were no significant differences between group A and group B in the prevalence of all complications (17.3% vs 14.0%, *P* = 0.479), periprosthetic fractures (3.2% vs 3.3% *P* = 0.945), dislocation (1.6% vs 4.1%, *P* = 0.224), or aseptic loosening (0.8% vs 0.8%, *P* = 0.973). The main type of nerve injury was lateral femoral cutaneous nerve injury in group A and sciatic nerve injury in group B. Lateral femoral cutaneous nerve injuries were identified in 7 (7/127, 5.5%) patients in group A, all of whom felt numbness of the anterolateral thigh; 5 of them recovered spontaneously within 1 year after surgery, and 2 of them still experienced numbness at the last follow up. Sciatic nerve injury was identified in 1 (1/121, 0.8%) patient in group B. This patient was symptomatic with limited ankle movement but gradually recovered during the first 3 months postoperatively. In this study, the prevalence of nerve injury in group A was significantly higher than that in group B (5.5% vs 0.8%, *P* = 0.037).

**Table 3:** Incidence of complications in the two groups

	Group A	Group B	*P*
Periprosthetic fracture	4 (3.2%)	4 (3.3%)	0.945
Dislocation	2 (1.6%)	5 (4.1%)	0.224
Nerve injury	7 (5.5%)	1 (0.8%)	0.037
Thigh pain	8 (6.3%)	6 (5.0%)	0.648
Aseptic loosening	1 (0.8%)	1 (0.8%)	0.973
Total	22 (17.3%)	17 (14.0%)	0.479

Note: Group A: DAA, group B: PA

We also analysed the Harris hip score (HHS) and FHJ-12 score at different time points in the two groups (Table 4). At 1 month after surgery, we found a significant difference between the two groups in the HHS (71.03 ± 8.04 in group A, 68.39 ± 8.37 in group B, *P* = 0.014) and FJS-12 score (50.78 ± 7.57 in group A, 47.68 ± 7.34 in group B, *P* = 0.001). At 1 year after surgery, there was no significant difference between the two groups in the HHS (89.80 ± 3.25 vs 89.92 ± 3.22, *P* = 0.812). The mean FJS-12 score in group A (68.78 ± 7.54) was higher than that in group B (58.84 ± 8.91) (*P* < 0.001). At 5 years after surgery, no significant differences were found between the two groups in either the HHS or FJS-12 score (92.88 ± 2.96 in group A and 92.93 ± 2.53 in group B, *P* = 0.888). The mean FJS-12 score in group A (73.38 ± 7.21) was higher than that in group B (67.16 ± 9.12) (P < 0.001).

**Table 4:** Harris hip score and FHJ-12 score of the two groups

	Harris hip score			FJS-12 score		
	Group A	Group B	*P*	Group A	Group B	*P*
1 month postoperatively	71.03 ± 8.04	68.39 ± 8.37	0.014	50.78 ± 7.57	47.68 ± 7.34	0.001
1 year postoperatively	89.80 ± 3.25	89.92 ± 3.22	0.812	68.78 ± 7.54	58.84 ± 8.91	< 0.001
5 years postoperatively	92.88 ± 2.96	92.93 ± 2.53	0.888	73.38 ± 7.21	71.87 ± 8.52	0.338

Note: Group A: DAA, group B: PA

### Multivariate linear regressions

In this study, a multiple linear regression model was used and adjusted to study the relationship between the FJS-12 score, HHS, demographic data (sex, age, BMI, smoking status, and alcohol consumption) and surgical approach. Post hoc analysis of the 1-month, 1-year, and 5-year postoperative FJS-12 scores using multiple linear regression analysis revealed that an excellent HHS led to good patient satisfaction at each time point. Every 1-point increase in the HHS of a patient led to an average increase of 0.536 points in the FJS-12 score at 1 month, a 0.431-point increase at 1 year postoperatively and a 0.468-point increase at 5 years postoperatively (Table 5). We found no differences in the FJS-12 scores at 1 month (B = -1.756, *P* = 0.028) and 1 year (B = -9.694, *P* < 0.001) between the 2 approaches. At 5 years postoperatively, the approach was not associated with a change in the FJS-12 score (B = -1.639, *P* = 0.112). Sex, age, BMI, smoking status, and alcohol consumption were not associated with a change in the FJS-12 score at any time point postoperatively.

**Table 5:** Multivariate linear regressions of the FJS scores

	1 month after surgery		1 year after surgery		5 years after surgery	
	B	*P*	B	*P*	B	*P*
Approach	-1.756	0.028	-9.694	< 0.001	-1.639	0.112
Sex	−1.198	0.232	−0.444	0.727	−0.420	0.738
Age	0.001	0.976	−0.011	0.802	−0.007	0.877
BMI	0.035	0.748	− 0.071	0.637	−0.064	0.665
HSS (at this time)	0.536	< 0.001	0.431	< 0.001	0.468	0.016
Smoking status	2.223	0.144	3.250	0.118	3.074	0.135
Alcohol consumption	−2.072	0.150	−1.817	0.354	−2.652	0.174

Note: Group A: DAA, group B: PA

## Discussion

The CFP stem is not commonly considered an anatomical stem. Several scholars believe that the curved, tapered and neck-preserving design may make implantation more difficult, and this stem is associated with a high complication rate. In this study, we compared the clinical and radiologic outcomes of primary THA with CFP stems using the DAA versus the PA, and the patient cohorts were similar; there was no selection bias, and the only variable that differed between groups was the operative approach. Most importantly, the results demonstrated that the early HHS and FJS-12 score of the patients who underwent THA via the DAA were significantly higher than those of the patients who underwent THA via the PA. Furthermore, there was no significant difference in the complication rate or radiological measurements between the DAA and PA groups except nerve injury.

The FJS-12 is a new type of patient-reported outcome assessment that can evaluate joint awareness or the ability of patients to forget about their case of joint arthroplasty in their everyday life and during recreational activity. It has been confirmed that the DAA can lead to improvements in the early postoperative recovery period, thus decreasing medical costs and increasing patient satisfaction [20]. Worse patient satisfaction (lower FJS-12 scores or HHSs) theoretically leads to a longer length of stay and higher medical costs. In our study, the HHS (*P* = 0.014) and FJS-12 score (*P* = 0.001) in the DAA group were higher than those in the PA group at 1 month postoperatively. This finding may suggest that although patients are able to perform certain motions, those who undergo THA via the PA have a higher likelihood of feeling the prosthesis within 1 month after surgery. This finding is consistent with the results of previous studies [21]. However, the difference in the HHS between the two groups disappeared gradually within 1 year after surgery (*P* = 0.812). However, the significant difference in the FJS-12 score between the two groups persisted (*P* < 0.001). The patients who underwent THA via the PA still had a higher level of awareness of their prosthesis, which influenced the FJS-12 score. Similar results have been reported by other researchers in studies on prosthesis implantation without femoral neck preservation. It is well established that the stable movement status of the hip joint is associated with the comfortable feelings of patients undergoing THA. As a consequence, the soft-tissue protection, especially the posterior soft-tissue, is crucial important during the THA surgery so that the patient is expected to have a stable hip joint postoperatively. This comfortable feeling, namely, the stable status of hip joint, is mainly determined by FJS-12 score, a scoring system reflected the patient’s “awareness” of hip prosthesis. For instance, Nakata et al. [16] reported that the integrity of gluteus maximus and iliotibial tract are necessary for the stability of the hip join. Furthermore, Agten et al. [22] reported that compared with DAA, PA decreased the stability of hip joint due to the damage of external rotator and gluteus maximus. This could answer the question why the early stage FJS-12 score in patients undergoing THA via PA is significantly lower than that in patients with DAA in this study. In contrast to our results, Vivek et al. [21] reported that at 1 and 1.75 years postoperatively, although the scores consistently trended in favour of the DAA cohort at all time points, no statistically significant differences in the FJS-12 score were found between the DAA group and the PA group. The authors considered that the approach is not the only factor affecting patient satisfaction and the FJS-12 score. Potential intraoperative factors, such as surgeon experience, skill, case difficulty and prosthesis design, can subsequently affect pain and the FJS-12 score. At 5 years after surgery, in the present study, no significant differences were found between the two groups in the HHS (*P* = 0.888) or FJS-12 score (*P* = 0.338). This finding may suggest that hip function, pain, and awareness of prosthesis by the patients were not affected by the approach during the medium- and long-term follow-up periods. We considered that the scar tissue between the piriformis and conjoined tendons was repaired after transection and achieved an orientation resembling the native tendon in most patients. Although the transected structures that were restored may be weaker than before surgery, their strength was enough to maintain good hip function and stability. This “feeling of stability” can be expressed as “awareness” and quantified by the FJS-12 [23]. In terms of the long-term results, previous studies have similarly shown that there are no significant differences in functional outcomes or awareness of the prosthesis by patients between the DAA and posterior approach [24, 25].

Post hoc analysis of the 1-month, 1-year, and 5-year postoperative FJS-12 scores using multiple linear regression analysis revealed that a lower HHS led to worse patient satisfaction at each time point. Moreover, the FJS-12 score was related to the approach at an early time point, which is consistent with the results we mentioned earlier. We considered that although the focuses of the HHS and FJS-12 scoring systems are inconsistent, there is some overlap between them. The surgical approach affects the patients’ awareness of the prosthesis in the early stage, and numerous factors other than the surgical approach, such as those related to patient preconditioning, pre-emptive analgesia, and the rehabilitation protocol, significantly impact the outcome of THA. All of these factors may ultimately play a role in patient satisfaction and thus affect the FJS-12 score and HHS.

In our study, the radiologic indicators (neck-shaft angle, coronal alignment, sagittal alignment, stress shielding, anteversion, neck-preserving ratio, acetabular anteversion, acetabular inclination angle, acetabular depth, anterior-posterior offset, lateral offset, difference in leg length) of the prosthesis position were not comparable between groups. However, unlike some previous studies demonstrating that the surgical approach had influence on the prosthetic position (Takada et al. [18] reported that a significant correlation was found between femoral anteversion and stem anteversion in both the supine and lateral groups and flexed implantation was more likely in the supine group), there was no influence on femoral prosthesis position between different surgical approaches in this study. This is because the femoral stem implanted in this study was a CFP stem, which is characterized by a subcapital osteotomy level and self-anteversion design (Fig. 1a, c). It can be implanted without over-lifting of the proximal femur and wide exposure during the surgical procedure. Therefore, the surgical approach did not seem to affect the position of the CFP prosthesis in this study. However, the preserved length is a crucial factor affecting the position of the prosthesis [14]. In the current study, both DAA and PA patients showed excellent imaging results, which indicates that CFP prostheses are appropriate for anterior approach surgery. However, it should be noted that when the acetabulum is exposed and the acetabular component is implanted, the preserved femoral neck can be occluded. Although malpositioning of the acetabular component did not occur in any patient in our study, occlusion is a potential risk factor for malpositioning of the acetabular component.

In our study, the nerve injury rate in the DAA group was significantly higher than that in the PA group. However, a higher nerve injury rate does not always yield a worse prognosis for patients [26]. In fact, all instances of lateral cutaneous nerve injury caused only numbness of the anterolateral thigh. No other symptoms were identified, and most notably, no muscle paralysis of the lower extremity was observed. Nevertheless, surgeons should focus on preventing nerve damage when DAA THA is performed. Unfortunately, when sciatic nerve injury occurs, it typically causes widespread muscle paralysis of the lower extremity, ultimately leading to severe sensory and motor dysfunction.

Fractures of the proximal femur associated with the approach have been well documented [27]. In our study, the fracture rates of the two groups were consistent and were in line with those reported in previous studies (3%). However, for CFP prostheses, the reported rates of periprosthetic fractures are inconsistent (range 0.5 to 10.7%). We believe that this inconsistency is due to the lack of proficiency in CFP prosthesis THA. Carmelo et al. [28] confirmed that standard straight stems are more prone to cortical perforation than CFP stems in DAA THA. During the operation, we noticed that the unique curved design of the CFP stem can make implantation into the medullary cavity easier. We thought this uniquely curved and continuously tapered design may prevent cortical perforation at the tip of the prosthesis.

In a study of 505 patients undergoing DAA THA, 7 patients (1.4%) underwent repeat surgery for periprosthetic infection [29]. Other studies [9, 30] have also reported periprosthetic infection rates ranging between 1 and 2% among patients who underwent anterior approach THA surgeries. In the current study, the infection rates (0.8%) of group A and group B were close to those reported. We consider that changing only the surgical approach does not affect the infection rate after THA. We prefer to associate the rate of infection with the surgical time and aseptic procedures.

In the current study, no significant difference was found between the two groups in the thigh pain rate. It has been confirmed that a lower neck-preserving ratio rather than the surgical approach with a CFP stem may contribute to stress being concentrated in the distal femoral cortex, which increases the intramedullary pressure. A meta-analysis [31] showed that the risk of chronic postsurgical pain after THA ranges from 7 to 23%, and persistent thigh pain after short stem THA might no longer be considered a simple transmission of nociception but rather a complex and multidimensional pain experience, implying that the CFP stem has a certain advantage in that aspect after surgery.

Several limitations of this study should be considered. First, previous studies have demonstrated that numerous factors other than the surgical approach, such as those related to patient preconditioning, pre-emptive analgesia, and the rehabilitation protocol, significantly impact the outcome of THA [6]. All of these factors may ultimately play a role in patient satisfaction and thus affect the FJS-12 score and HHS. Second, the number of patients in each study group was small, leading to a low statistical power. However, patients were selected carefully because contralateral hip joint pain, limited range of motion, and the presence of a prosthetic joint may be confounding factors, as the presence of a pathology in the contralateral hip joint has been reported to influence patient-reported evaluations. Thus, we cannot determine the clinical significance of the differences between groups. Finally, we investigated clinical and radiological outcomes only 5 years after surgery. Significant differences may appear within such a period. However, the long-term outcomes between the two approaches in CFP THA remain unknown.

## Conclusions

In summary, unlike a “standard” femoral stem, whose alignment might be affected by surgical approach, alignment of the CFP stem is independent from the surgical approach. Regardless of the higher nerve injury rate, nerve injury from the DAA did not typically cause severe dysfunction of the lower extremity. Therefore, decisions regarding the surgical approach in patients undergoing THA with CFP stems can be made primarily based on the preference of the surgeon.

## Data Availability

All data generated or analysed during this study are included in this published article.
